# Potential predatory and legitimate biomedical journals: can you tell the difference? A cross-sectional comparison

**DOI:** 10.1186/s12916-017-0785-9

**Published:** 2017-03-16

**Authors:** Larissa Shamseer, David Moher, Onyi Maduekwe, Lucy Turner, Virginia Barbour, Rebecca Burch, Jocalyn Clark, James Galipeau, Jason Roberts, Beverley J. Shea

**Affiliations:** 1 Centre for Journalology, Clinical Epidemiology Program, Ottawa Hospital Research Institute, Ottawa, K1H 8L6 Canada; 2 School of Epidemiology, Public Health and Preventative Medicine, University of Ottawa, Ottawa, K1H 8M5 Canada; 3 School of Medicine, Dentistry and Biomedical Sciences, Queen’s University Belfast, Belfast, BT9 7BL UK; 4 Clinical Epidemiology Program, Ottawa Hospital Research Institute, Ottawa, K1H 8L6 Canada; 5 Office of Research Ethics and Integrity, Queensland University of Technology (QUT), Brisbane, QLD 4000 Australia; 6 Brigham and Women’s Hospital, Harvard Medical School, Boston, 02115 USA; 7 icddr,b, Dhaka, 1000 Bangladesh; 8 Origin Editorial, Plymouth, MA 02360 USA; 9 Knowledge Synthesis Group, Clinical Epidemiology Program, Ottawa Hospital Research Institute, Ottawa, K1H 8L6 Canada

**Keywords:** Predatory, Open access, Scientific publishing, Publishing models, Biomedical journal, Journalology

## Abstract

**Background:**

The Internet has transformed scholarly publishing, most notably, by the introduction of open access publishing. Recently, there has been a rise of online journals characterized as ‘predatory’, which actively solicit manuscripts and charge publications fees without providing robust peer review and editorial services. We carried out a cross-sectional comparison of characteristics of potential predatory, legitimate open access, and legitimate subscription-based biomedical journals.

**Methods:**

On July 10, 2014, scholarly journals from each of the following groups were identified – potential predatory journals (source: Beall’s List), presumed legitimate, fully open access journals (source: PubMed Central), and presumed legitimate subscription-based (including hybrid) journals (source: Abridged Index Medicus). MEDLINE journal inclusion criteria were used to screen and identify biomedical journals from within the potential predatory journals group. One hundred journals from each group were randomly selected. Journal characteristics (e.g., website integrity, look and feel, editors and staff, editorial/peer review process, instructions to authors, publication model, copyright and licensing, journal location, and contact) were collected by one assessor and verified by a second. Summary statistics were calculated.

**Results:**

Ninety-three predatory journals, 99 open access, and 100 subscription-based journals were analyzed; exclusions were due to website unavailability. Many more predatory journals’ homepages contained spelling errors (61/93, 66%) and distorted or potentially unauthorized images (59/93, 63%) compared to open access journals (6/99, 6% and 5/99, 5%, respectively) and subscription-based journals (3/100, 3% and 1/100, 1%, respectively). Thirty-one (33%) predatory journals promoted a bogus impact metric – the Index Copernicus Value – versus three (3%) open access journals and no subscription-based journals. Nearly three quarters (*n* = 66, 73%) of predatory journals had editors or editorial board members whose affiliation with the journal was unverified versus two (2%) open access journals and one (1%) subscription-based journal in which this was the case. Predatory journals charge a considerably smaller publication fee (median $100 USD, IQR $63–$150) than open access journals ($1865 USD, IQR $800–$2205) and subscription-based hybrid journals ($3000 USD, IQR $2500–$3000).

**Conclusions:**

We identified 13 evidence-based characteristics by which predatory journals may potentially be distinguished from presumed legitimate journals. These may be useful for authors who are assessing journals for possible submission or for others, such as universities evaluating candidates’ publications as part of the hiring process.

## Background

The Internet has transformed scholarly publishing. It has allowed for the digitalization of content and subsequent online experimentation by publishers, enabling print journals to host content online, and set the course for online open-access publishing. Nevertheless, an unwelcome consequence of the Internet age of publishing has been the rise of so-called predatory publishing.

In the traditional subscription model of publishing, journals typically require transfer of copyright from authors for articles they publish and their primary revenue stream is through fees charged to readers to access journal content, typically subscription fees or pay-per-article charges. Open access publishing, in contrast, typically allows for authors to retain copyright, and is combined with a license (often from Creative Commons), which enables free and immediate access to published content coupled with rights of reuse [1]. Some open access journals [2] and many hybrid journals (i.e., those with some open access content and also with non-open access content) [3] use a business model that relies upon publication charges (often called article publication or processing charges, or APC) to the author or funder of the research to permit immediate and free access.

Predatory publishing is a relatively recent phenomenon that seems to be exploiting some key features of the open access publishing model. It is sustained by collecting APCs that are far less than those found in presumably legitimate open access journals and which are not always apparent to authors prior to article submission. Jeffrey Beall, a librarian at the University of Colorado in Denver, first sounded the alarm about ‘predatory journals’ and coined the term. He initiated and maintains a listing of journals and publishers that he deems to be potentially, possibly, or probably predatory, called Beall’s List [4] (content unavailable at the time of publishing)﻿﻿. Their status is determined by a single person (Jeffrey Beall), against a set of evolving criteria (in its 3rd edition at the time of writing) that Beall has based largely on The Committee On Publication Ethics (COPE) Code of Conduct for Journal Editors and membership criteria of the Open Access Scholarly Publisher’s Association [5–7]. Others have suggested similar criteria for defining predatory journals [8, 9].

The phenomenon of predatory publishing is growing and opinions on its effects are divided. Critics say that it is extremely damaging to the scientific record and must be stopped [10, 11]. Others feel that, while problematic, predatory publishing is a transient state in publishing and will disappear or become obvious over time [12]. A fundamental problem of predatory journals seems to be that they collect an APC from authors without offering concomitant scholarly peer review (although many claim to [13]) that is typical of legitimate journals [14]. Additionally, they do not appear to provide typical publishing services such as quality control, licensing, indexing, and perpetual content preservation and may not even be fully open access. They tend to solicit manuscripts from authors through repeated email invitations (i.e., spam) boasting open access, rapid peer review, and praising potential authors as experts or opinion leaders [13]. These invitations may seem attractive or an easy solution to inexperienced or early career researchers who need to publish in order to advance their career, or to those desperate to get a publication accepted after a number of rejections, or to those simply not paying attention. Predatory journals may also be a particular problem in emerging markets of scientific research, where researchers face the same pressure to publish, but lack the skills and awareness to discern legitimate journals from predatory ones.

Still, many researchers and potential authors are not aware of the problem of predatory journals and may not be able to detect a predatory journal or distinguish one from a legitimate journal. In order to assist readers, potential authors, and others in discerning legitimate journals from predatory journals, it would be useful to compare characteristics from both predatory and non-predatory journals to see how they differ.

In this study, we undertook a cross-sectional study comparing the characteristics of three types of biomedical journals, namely (1) potential predatory journals, (2) presumed legitimate, fully open access journals, and (3) presumed legitimate subscription-based biomedical journals that may have open access content (e.g., hybrid).

## Methods

### Design

This was a cross-sectional study.

### Journal identification and selection

We searched for journals on July 10, 2014. For feasibility, only journals with English-language websites were considered for inclusion and we set out to randomly select 100 journals within each comparison group. The following selection procedures were used to identify journals within each comparison group:

- ***Potential predatory journals (‘Predatory’):*** We considered all journals named on Beall’s List of single publishers for potential inclusion. We applied the MEDLINE Journal Selection criteria [15]: “[Journals] *predominantly devoted to reporting original investigations in the biomedical and health sciences, including research in the basic sciences; clinical trials of therapeutic agents; effectiveness of diagnostic or therapeutic techniques; or studies relating to the behavioural, epidemiological, or educational aspects of medicine.*” Three independent assessors (OM, DM, LS) carried out screening in duplicate. From the identified biomedical journals, a computer-generated random sample of 100 journals was selected for inclusion. Journals that were excluded during data extraction were not replaced.
- ***Presumed legitimate fully open-access journals (‘Open Access’):*** A computer-generated, random sample of 95 journals from those listed on PubMed Central as being full, immediate open access, were included. In addition, five well-established open access journals were purposefully included: *PLOS Medicine*, *PLOS One*, *PLOS Biology*, *BMC Medicine*, and *BMC Biology*.
- ***Presumed legitimate subscription-based journals (‘Subscription-based’):*** A computer-generated, random sample of 100 journals from those listed in the Abridged Index Medicus (AIM) was included. AIM was initiated in 1970 containing a selection of articles from 100 (now 119) English-language journals, as a source of relevant literature for practicing clinicians [16]. AIM was used here since all journals in this group were initiated prior to the digital era and presumed to have a maintained a partially or fully subscription-based publishing model [confirmed by us].

For all journals, their names and URLs were automatically obtained during the journal selection process and collected in Microsoft Excel. Screening and data extraction were carried out in the online study management software, Distiller SR (Evidence Partners, Ottawa, Canada). Journals with non-functioning websites at the time of data extraction or verification were excluded and not replaced.

### Data extraction process

Data were extracted by a single assessor (OM) between October 2014 and February 2015. An independent audit (done by LS) of a random 10% of the sample showed discrepancies in 34/56 items (61%) on at least one occasion. As such, we proceeded to verify the entire sample by a second assessor. Verification was carried out in April 2015 by one of eight assessors (RB, JC, JG, DM, JR, LS, BJS, LT) with experience and expertise on various aspects of biomedical publishing process. Any disagreements that arose during the verification process were resolved by third party arbitration (by LS or LT). It was not possible to fully blind assessors to study groups due to involvement in the journal selection process (OM, DM, LS).

### Data extraction items

Items for which data were extracted were based on a combination of items from Beall’s criteria (version 2, December 2012) for determining predatory open-access publishers [6], the COPE Code of Conduct for Journal Publishers (http://publicationethics.org/resources/code-conduct), and the OASPA Membership criteria (http://oaspa.org/membership/membership-criteria/). Data for 56 items were extracted in the following nine categories: aims and scope, journal name and publisher, homepage integrity (look and feel), indexing and impact factor, editors and staff, editorial process and peer review, publication ethics and policies, publication model and copyright, and journal location and contact.

### Data analysis

Data were descriptively summarized within each arm. Continuous data were summarized by medians and interquartile range (IQR); dichotomous data were summarized using proportions.

## Results

**Fig. 1:**
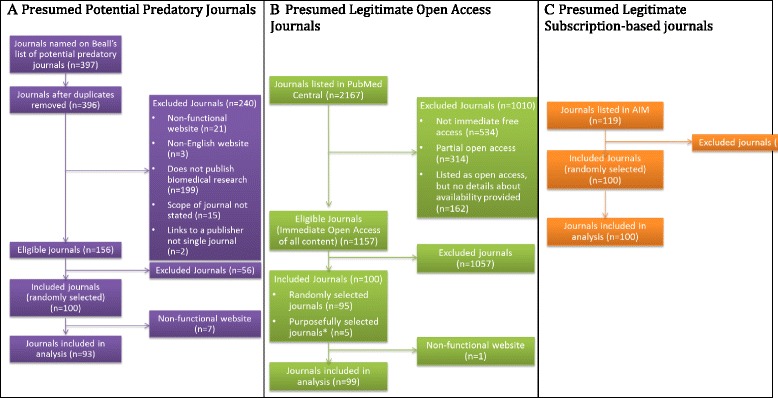
Flow diagram of journal identification, selection, and inclusion in each study group. **a** Potential predatory journals identified from Beall’s list. **b** Presumed legitimate fully open access journals identified from PubMed Central including five purposely selected journals: *PLOS Medicine*, *PLOS One*, *PLOS Biology*, *BMC Medicine*, and *BMC Biology*. **c** Subscription-based journals identified from AIM

Ninety-three potential predatory journals, 99 open access journals, and 100 subscription-based journals were included in the analysis. The process of journal identification, inclusion, and exclusions within each study group is outlined in Fig. 1; 397 journals were identified as potential predatory journals. After de-duplication and screening for journals publishing biomedical content, 156 journals were identified, from which a random sample of 100 were chosen. Seven journals from the predatory group and one from the legitimate open access group were excluded during data extraction due to non-functional websites. No journal appeared in more than one study group.

There were four unanticipated journal exclusions during data extraction in the presumed legitimate open access and subscription-based groups for which randomly selected replacement journals were used. One journal was listed twice in the open access group and was deemed to be a magazine rather than a scientific journal. Two journals in the subscription-based journal group were deemed to be a magazine and a newsletter, respectively. The decision to exclude and replace these was made post-hoc, by agreement between LS and DM.

Our main findings of journal characteristics for each data extraction category are summarized in Tables 1, 2, 3, 4, 5, 6, 7, 8 and 9.

**Table 1:** Aims and scope

		Predatory, *N* = 93, *n* (%)	Open Access, *N* = 99, *n* (%)	Subscription-based, *N* = 100, *n* (%)
General research area	Biomedical only	47 (50.53)	99 (100)	100 (100)
	Biomedical & non-biomedical	46 (49.46)	N/A	N/A^a^
Medical subject categories	Top 5 listed (n)	Pharmacology/Toxicology (59) Research/Laboratory Medicine & Medical Technology (37) Immunology (22) Nursing (18) Dentistry (17)	Research/Laboratory Medicine & Medical Technology (28) Immunology (20) Pharmacology/Toxicology (13) Nursing (12) General and Internal Medicine (12)	General and Internal Medicine (15) Surgery (13) Cardiac & Cardiovascular Systems (12) Public, Environmental & Occupational Health (10) Endocrinology, Metabolism & Nutrition (9)
Number of medical categories^b^	Median (IQR)	2 (1–4) (Range 1–31)	2 (1–2) Range (1–23)	1 (1–2) Range (1–16)

^a^Not assessed; presumed biomedical due to source

^b^Number of journals providing this information: Predatory, *n* = 86; Open Access, *n* = 94; Subscription, *n* = 99

**Table 2:** Journal name and publisher

		Predatory, *N* = 93, *n* (%)	Open Access, *N* = 99, *n* (%)	Subscription-based, *N* = 100, *n* (%)
Journal name similar to another journal	Yes	51 (54.84)	17 (17.17)	22 (22.00)
Journal URL reflects journal name	Yes	84 (90.32)	95 (95.96)	94 (93.49)
Journal name represents aims and scope	Yes	82 (88.17)	96 (96.97)	95 (95.00)
	No	7 (7.53)	2 (2.02)	1 (1.00)
	Aims/scope not stated	4 (4.30)	1 (1.01)	4 (4.00)
Publisher name indicated	Yes	49 (52.69)	99 (100.00)	98 (98.00)
	No	44 (47.31)	0 (0)	2 (2.00)
Publisher name^a^	Top 5 listed (*n*)	Laxmi Book Publications (2) *All other publishers publish a single journal*	BioMed Central (27) Hindawi (14) Medknow (6) Libertas Academica (4) PLOS and OceanSide Publications (3 each)	Elsevier (29) Lippincott Williams & Wilkins (14) BMJ (6) Wiley (6) Oxford University Press (6)
Publisher URL provided^b^	Yes	16/49 (32.65)	86/99 (86.87)	97/98 (98.98)
	No, found using Google	11/33 (33.33)	6/13 (46.15)	0/1 (0.00)
	No, not found on Google	22/33 (66.67)	7/13 (53.87)	1/1 (100.00)
Publisher/owner listed as Editor-in-Chief^c^	Yes	6/37 (16.22)	1/82 (1.22)	0/89 (0)
	No	23/37 (62.16)	81/82 (98.78)	89/89 (100)
	Cannot tell	8/37 (21.62)	0/82 (0)	0/89 (0)

^a^Data presented for journals where publisher was identified

^b^Denominator of fractions indicates the number of journals where the variable concerned was relevant

^c^Denominator is the number journals where both publisher and Editor-in-Chief names were provided

**Table 3:** Location and contact information

		Predatory, *N* = 93, *n* (%)	Open Access, *N* = 99, *n* (%)	Subscription-based, *N* = 100, *n* (%)
Country name in journal title differs from country listed in ‘contact us’^a^	Yes	11/21 (52.38)	4/13 (30.77)	1/31 (3.23)
Country named in contact address^b^	Top 5 listed (*n*)^e^	India (40) UK (5) USA (4) Romania (3) Bulgaria (2)	UK (34) South Korea (9) Iran (5) New Zealand (4) Germany (3)	USA (66) UK (16) Australia (1) Canada (1) New Zealand (1)
Addresses in low/low- to middle-income countries^f^		48/64 (75.00%)	18/92 (19.56%)	0/83 (0.00%)
‘Contact us’ mechanism	Email address	90 (96.77)	91 (91.92)	87 (87.00)
	Fillable web form	42 (45.16)	22 (22.22)	25 (25.00)
	No contact apparent	1 (1.08)	1 (1.01)	7 (7.00)
Non-professional email address provided (e.g., Yahoo, Google, AOL)^c^	Yes	57/90 (63.33)	9/91 (9.89)	5/87 (5.75)
Email address provided for Editor-in-Chief^d^	Yes	39/71 (54.93)	23/82 (28.05)	29/91 (31.87)

^a^Denominator of fraction represents number of journals naming a country in the title

^b^More than one country named by some journals

^c^Denominator of fractions indicates the number of journals where the variable concerned was relevant

^d^Denominator of fractions indicates the number of journals where an Editor-in-Chief was listed

^e^Number of journals providing this information: Predatory, *n* = 64; Open access *n* = 92; Subscription, *n* = 83

^f^Categorized using 2014 World Bank Data: http://data.worldbank.org/about/country-and-lending-groups

**Table 4:** Homepage integrity (look and feel)

		Predatory, *N* = 93, *n* (%)	Open Access, *N* = 99, *n* (%)	Subscription-based, *N* = 100, *n* (%)
Presence of spelling and grammatical errors	Yes	61 (65.59)	6 (6.06)	3 (3.00)
Colloquialisms/slang used	Yes	2 (2.15)	1 (1.01)	0 (0)
Presence of distorted/unauthorized images	Yes	59 (63.44)	5 (5.05)	1 (1.00)
Type of user targeted by homepage language	Readers	3 (3.23)	14 (14.14)	58 (58.00)
	Authors	46 (49.46)	13 (13.13)	2 (2.00)
	Both	28 (30.11)	51 (51.51)	26 (26.00)
	Cannot tell	16 (17.20)	21 (21.21)	14 (14.00)

**Table 5:** Indexing and impact factor

		Predatory, *N* = 93, *n* (%)	Open Access, *N* = 99, *n* (%)	Subscription-based, *N* = 100, *n* (%)
Indicate having Thomson ISI ‘Journal Impact Factor’ (JIF)	Yes	21 (22.58)	38 (38.38)	80 (80.00)
	If yes, median JIF (IQR)^a^	2.958 (0.500–3.742)	1.750 (1.330–2.853)	4.275 (2.469–6.239)
Other journal-level metric indicated	Yes	54 (58.06)	16 (16.16)	62 (62.00)
	Number of other metrics, median (IQR)	2 (1–2)	1 (1–2)	1 (1–2)
Other metric	Top 5 listed (*n*)	Index Copernicus Value (31) Global Impact Factor (9) Scientific Journal Impact Factor (9) Scientific Journal Rankings/SciMago/Scopus (7) Total citations (7)	Scientific Journal Rankings/SciMago/Scopus (6) Total citations (5) Index Copernicus Value (3) h-index (2) 5-year impact factor (2)	5-year impact factor (27) Overall ranking (15) Scientific Journal Rankings/SciMago/Scopus (8) Source Normalized Impact Factor (8) Eigenfactor (7)
Databases indexed/listed	PubMed MEDLINE CINAHL EMBASE PsycInfo Google Scholar Other	6 (6.65) 2 (2.15) 0 (0) 14 (15.05) 0 (0) 47 (50.54) 83 (89.25)	85 (85.86) 21 (21.21) 4 (4.04) 42 (42.42) 7 (7.07) 59 (59.60) 94 (94.95)	17 (17.00) 39 (39.00) 15 (15.00) 32 (32.00) 6 (6.00) 1 (1.00) 55 (55.00)
DOAJ mentioned (indexed or applying for indexing)	Yes	48 (51.61)	65 (65.65)	1 (1.00)
ISSN found/identified	Yes	91 (97.85)	95 (95.96)	72 (72.00)
Editorial organizations mentioned	ICMJE WAME CSE EASE OASPA Other^b^ None	16 (17.2) 4 (4.30) 0 (0) 0 (0) 0 (0) 2 (2.15) 70 (75.27)	79 (79.8) 8 (8.08) 2 (2.02) 2 (2.02) 6 (6.06) 7 (7.07) 10 (10.10)	74 (74.00) 14 (14.00) 2 (2.00) 0 (0) 1 (1.00) 3 (3.00) 20 (20.00)

*ICMJE* International Committee of Medical Journal Editors, *WAME* World Association of Medical Editors, *CSE* Council of Science Editors, *EASE* European Association of Science Editors

^a^Number of journals providing this information: Predatory, *n* = 21; Open Access, *n* = 37; Subscription, *n* = 80

^b^Other: Association of Learned and Professional Society Publishers, Council of Biology editors, European Medical Writers Association, Higher Attestation Commission of the Russian Ministry of Education and Science, International Association of Scientific, Technical, & Medical Publishers, Korean Association of Medical Journal Editors, OAIster - The Open Access Initiative

**Table 6:** Editors and staff

		Predatory, *N* = 93, *n* (%)	Open Access, *N* = 99, *n* (%)	Subscription-based, *N* = 100, *n* (%)
Named Editor-in-Chief	Yes	71 (76.34)	82 (82.83)	91 (91.00)
Formal editorial board named	Yes	60 (64.52)	92 (92.93)	72 (72.00)
	If yes, number of members (median, IQR)	23 (14–37)	32.5 (22–50)	27.5 (16.5–62)
Composition of journal staff	Managing/handling editor	22 (23.66)	18 (18.18)	41 (41.00)
	Associate editor	30 (32.26)	47 (47.47)	68 (68.00)
	Academic editor	0 (0)	0 (0)	1(1.00)
	Statistical editor	2 (2.15)	4 (4.04)	20 (20.00)
	Editorial staff	8 (8.60)	7 (7.07)	19 (19.00)
	Other^a^	43 (46.24)	45 (45.45)	75 (75.00)
	None of the above	26 (27.96)	24 (24.24)	3 (3.00)
Validity check^b,c^	Legitimate	24/90 (26.67)	95/98 (96.94)	97/97 (100.00)
	False/made up	41/90 (45.56)	2/98 (2.04)	1/97 (1.03)
	Used without permission	66/90 (73.33)	2/98 (2.04)	1/97 (1.03)
Institutional affiliation indicated^c^	Editor-in-Chief	40/71 (56.33)	71/82 (86.59)	57/91 (62.64)
	Editors/staff	42/67 (62.69)	56/75 (74.67)	48/97 (49.48)
	Editorial board members	48/60 (80.00)	81/92 (88.04)	31/72 (43.06)

^a^163 different terms were described, e.g., Editorial office, co-editors, editor(s), deputy editors, acting editor, acting deputy editor, assistant managing editor

^b^Assessors were asked to perform a Google search of the Editor-in-Chief and two other randomly selected editors/staff/board members along with their affiliation (if provided) and make a subjective assessment of whether the names appear to be legitimate, false/made up, used without permission. Assessments were based on searches through online profiles (i.e., LinkedIn, faculty bio, etc.) for mention of journal affiliation; categories not distinct since judgments based on multiple editors

^c^Denominator of fractions indicates the number of journals where the variable concerned was relevant

**Table 7:** Editorial process and peer review

		Predatory, *N* = 93, *n* (%)	Open Access, *N* = 99, *n* (%)	Subscription-based, *N* = 100, *n* (%)
Stated manuscript handling process	Yes	53 (56.99)	90 (90.91)	86 (86.00)
Submission system	Third party	2 (2.15)	26 (26.26)	75 (75.00)
	Journal-specific system	47 (50.54)	70 (70.71)	21 (21.00)
	Emailed to journal	65 (69.89)	2 (2.02)	3 (3.00)
	Other^a^	2 (2.15)	5 (5.05)	0 (0)
	Not found	0 (0)	0 (0)	3 (3.00)
States using peer review	Yes	89 (95.70)	99 (100)	92 (92.00)
Indicated processing time	‘Rapid’ publication	38 (40.86)	16 (16.16)	9 (9.00)
	<1 week peer review turnaround	17 (18.28)	3 (3.03)	1 (1.00)
	Expedited peer review	9 (9.68)	4 (4.04)	7 (7.00)
	Not indicated	47 (50.54)	84 (84.85)	85 (85.00)

^a^Other: mailed to journal, publisher-specific system

**Table 8:** Publication ethics and policies

		Predatory, *N* = 93, *n* (%)	Open Access, *N* = 99, *n* (%)	Subscription-based, *N* = 100, *n* (%)
COPE mentioned	Yes	13 (13.98)	77 (77.78)	33 (33.00)
Publication ethics technologies:	ORCID	2 (2.15)	9 (90.91)	3 (3.00)
	Crossref	10 (10.75)	23 (23.23)	7 (7.00)
	Crossmark	0 (0)	1 (1.01)	2 (2.00)
	Crosscheck/iThenticate	1 (1.08)	57 (57.58)	16 (16.00)
	none	81 (87.10)	40 (40.40)	77 (77.00)
Retraction policy	Yes	12 (12.90)	44 (44.44)	68 (68.00)
Corrections or errata policy	Yes	22 (23.66)	50 (50.51)	50 (50.00)
Plagiarism policy	Yes	44 (47.31)	70 (70.71)	49 (49.00)
Instructions to authors available	Yes	90 (96.77)	98 (98.99)	97 (97.00)
If yes, manuscript preparation guidance^a^	Yes	86/90 (95.56)	98/98 (100)	97/97 (100.00)
If yes, reporting guideline(s) mentioned^a^	EQUATOR	0/90 (0.00)	25/98 (25.25)	24/97 (24.00)
	CONSORT	4/90 (4.44)	37/98 (37.76)	57/97 (58.76)
	PRISMA	1/90 (1.11)	26/98 (26.53)	32/97 (32.99)
	STROBE	1/90 (1.11)	27/98 (27.55)	23/97 (23.71)
	STARD	1/90 (1.11)	29/98 (29.59)	22/97 (22.68)
	Other^b^	2/90 (2.22)	30/98 (30.61)	27/97
	None	85/90 (91.40)	54/98 (54.55)	35/97 (35.00)
Study registration required	Yes	6 (6.45)	56 (56.57)	62 (62.00)

*ORCID* Open Researcher and Contributor ID

^a^Denominator of fractions indicates the number of journals where the variable concerned was relevant

^b^21 other reporting guidelines mentioned, including ARRIVE, CARE, CHEERS, COREQ, ENTREQ, HuGENet, MIAME, MIBBI, MOOSE, QUOROM, ORION, PRISMA-P, RATS, REDHOT, REFLECT, SPIRIT, SQUIRE, STREGA, TREND, TRIPOD, a custom journal checklist

**Table 9:** Publication model and copyright

		Predatory, *N* = 93, *n* (%)	Open Access, *N* = 99, *n* (%)	Subscription-based, *N* = 100, *n* (%)
Mentions digital preservation of content	Yes	6 (6.45)	46 (46.46)	30 (30.00)
Claims to be Open Access	Yes	83 (89.25)	94 (94.95)	0 (0)
	No	10 (10.75)	5 (5.05)	39 (39.00)
	Partial (some content)	0	0	42 (42.00)
Number of apparent revenue sources	None apparent	14 (15.05)	8 (8.08)	2 (2.00)
	1	62 (66.67)	52 (50.51)	17 (17.00)
	2	16 (17.20)	27 (27.27)	40 (40.00)
	3	1 (1.08)	7 (7.07)	27 (27.00)
	4	0 (0)	5 (5.05)	14 (14.00)
Type of revenue source	Article processing charge	73 (78.49)	74 (74.75)	60 (60.00)
	Submission fee	0 (0)	2 (2.02)	3 (3.00)
	Subscription fee	13 (13.98)	26 (28.28)	95 (95.00)
	Reprints	5 (5.38)	28 (28.28)	42 (42.00)
	Advertisements	3 (3.23)	10 (11.11)	27 (26.00)
	Cannot tell	15 (16.13)	8 (8.08)	4 (4.00)
	Other^a^	3 (3.23)	7 (7.07)	7 (7.00)
APC (USD 04/2015)	Total indicating^b^	59/73 (80.82)	70/74 (94.59)	44/60 (73.33)
	Amount (Median [IQR])^c^	100 (63–150)	1866 (800–2205)	3000 (2500–3000)
	Not stated/found	11 (15.07)	0 (0)	8 (13.11)
	Difficult to find	13 (17.81)	2(2.78)	12 (19.67)
Copyright retention	Total indicating	75 (80.65)	94 (94.95)	87 (87.00)
	Author retains	9 (12.00)	64 (68.09)	32 (36.78)
	Journal/publisher retains	66 (88.00)	28 (29.79)	54 (62.07)
	Other	0 (0)	2 (2.02)	1 (1.00)
	Not found/reported	18 (19.35)	5 (5.05)	13 (13.00)
Creative Commons mentioned	Total indicating	22 (23.66)	89 (89.90)	43 (43.00)
	Attribution (CC BY)	12/22 (54.55)	62/89 (69.66)	21/43 (48.84)
	Attribution-ShareAlike (CC BY-SA)	0/22 (0)	1/89 (1.12)	0/43 (0)
	Attribution-NoDerivs (CC BY-ND)	0/22 (0)	1/89 (1.12)	0/43 (0)
	Attribution-Non-Commercial (CC BY-NC)	2/22 (9.09)	19/89 (21.35)	17/43 (39.53)
	Attribution-Non-Commercial-ShareAlike (CC BY-NC-SA)	4/22 (18.18)	7/89 (7.87)	6/43 (13.95)
	Attribution-Non-Commercial-NoDerivs (CC BY-NC-ND)	3/22 (13.64)	2/89 (2.25)	30/43 (69.77)
	No specific license indicated	1/22 (4.55)	0 (0)	0 (0)

^a^Other: per page fee, per author fee, per colour figure fee, and other one-time publication fee

^b^Denominator of fractions indicates the number of journals where an article processing charge (APC) was specifically identified

^c^Data presented for journals indicating an APC

### Homepage and general characteristics

About half of the predatory journals in our sample indicated interest in publishing non-biomedical topics (e.g., agriculture, geography, astronomy, nuclear physics) alongside biomedical topics in the stated scope of the journal and seemed to publish on a larger number of topics than non-predatory journals (Table 1). Predatory journals included pharmacology and toxicology (*n* = 59) in the scope of their journal four and a half times more often than open access journals (*n* = 13) and almost 30 times more than subscription-based journals (*n* = 2).

When we examined the similarity of the journal name to other existing journals (e.g., one or two words different on the first page of Google search results), we found that over half of predatory journals (*n* = 51, 55.84%) had names that were similar to an existing journal compared to only 17 open access journals (17.17%) and 22 subscription-based journals (22.00%) (Table 2). In all study groups, the journal name was well reflected in the website URL. For journals that named a country in the journal title, some journals named a different country in the journal contact information (11/21 (52.38%) predatory; 4/13 (30.77%) open access; 1/31 (3.23%) subscription-based) (Table 3). There was a high prevalence of predatory journals from low or low- to middle-income countries (LMICs) (48/64, 75.00%) compared to open access journals (18/92, 19.56%); none of the subscription-based journals listed LMIC addresses.

We assessed the integrity of the homepage by examining the content for errors (Table 4). Spelling and grammatical errors were more prevalent in predatory journals (*n* = 61, 65.59%) compared to in open access (*n* = 6, 6.06%) and subscription-based journals (*n* = 3, 3.00%). In addition, we found a higher frequency of distorted or potentially unauthorized image use (e.g., company logos such as Google, MEDLINE, COPE, Crossref) in predatory journals (n = 59, 63.44%) versus in open access (*n* = 5, 5.05%) and subscription-based journals (*n* = 1, 1%). Readers were the main target of language used on subscription-based journal webpages (*n* = 58, 58%) but less so in open access (*n* = 14, 14.14%) and predatory (*n* = 3, 3.23%) journals, where authors (predatory journals) or both authors and readers (open access journals) were the primary target.

### Metrics and indexing

Most subscription-based journals indicated having a journal impact factor (assumed 2-year Thomson Reuters JIF unless otherwise indicated) (*n* = 80, median 4.275 (IQR 2.469–6.239)) compared to less than half of open access journals (*n* = 38, 1.750 (1.330–2.853)) and fewer predatory journals (*n* = 21, 2.958 (0.500–3.742)) (Table 5). More than half of predatory journals (*n* = 54, 58.06%) and subscription-based journals (*n* = 62, 62%) mentioned another journal-level metric, compared to only 16 (16.16%) open access journals. A metric called the Index Copernicus Value was the most common other metric mentioned in 31 predatory journals (33.33%) and in three open access journals (3.03%), followed by the 5-year impact factor (Thomson Reuters) mentioned in two open access journals (2.02%) and 27 subscription-based journals (27.00%), followed by the Scientific Journal Rankings (i.e., SCImago Journal Rank by Scopus) mentioned in seven predatory, six open access, and eight subscription-based journals. The top databases in which journals indicated being indexed were Google Scholar for predatory journals (*n* = 47, 50.54%), PubMed for open access journals (*n* = 85, 85.86%), and MEDLINE for subscription-based journals (*n* = 39, 39%). About half of predatory journals (*n* = 48, 51.61%) and 65 (65.65%) open access journals mention DOAJ (indexed in or applied for indexing). International Committee of Medical Journal Editors (ICMJE) was mentioned in some capacity in 16 predatory journals and about three quarters of non-predatory journals.

### Editors and editorial process

Nearly a quarter (*n* = 22, 23.66%) of predatory journals, 17 (17.17%) open access journals, and 9 (9%) subscription-based journals did not name an editor-in-chief (EIC) (Table 6). Of those that did, 40 (56.33%) predatory, 71 (86.59%) open access, and 57 (62.64%) subscription-based journals provided an institutional affiliation for the named EIC. An editorial board listing individual members was provided in 60 (64.52%) predatory journals, 92 (92.93%) open access journals, and 72 (72%) subscription-based journals, each comprising a median of 23 (IQR 14–37), 32.5 (22–50), and 27.5 (16.5–62) board members, respectively. If editors, journal staff, or editorial board members were identified, we completed a subjective assessment of the validity of three arbitrary names and the likelihood of their association with the journal by performing a Google search of their name (in quotations) and searching any online profiles for affiliation with the journal. Details of this assessment can be found in Table 6. For journals with names of editors, staff, or board members available, 100% of names checked in subscription-based journals were found to be legitimate as well as in 95/98 (96.94%) open access journals. Only 24/90 (26.67%) named editors, staff, or board members were assessed as having a legitimate association with the journal among predatory journals. Almost 100% of non-predatory journals appear to use a manuscript submission system, whereas just over half of predatory journals use such a system; almost 70% of predatory journals request that authors send their manuscripts by email and 63% of those journals provide what appears to be a non-professional (e.g., Gmail, Yahoo) email address to do so. Almost all journals (95% predatory journals, 100% open access journals, 92% of subscription-based journals) indicate using peer review during publication consideration (Table 7).

### Publication ethics and policies

We examined journals’ promotion and practices around publications ethics (Table 8). About three quarters (*n* = 77, 77.78%) of open access journals and about a third (*n* = 33, 33.00%) of subscription-based journals mentioned COPE somewhere on their website whereas only 13 predatory journals (13.98%) did. Few predatory journals had policies about retractions (*n* = 12, 12.90%), corrections/errata (*n* = 22, 23.66%), or plagiarism (*n* = 44, 47.31%) whereas more than half of all non-predatory journals had available policies for all three (retractions: *n* = 112, 56.28%; corrections/errata: *n* = 100, 50.25%; plagiarism: *n* = 199, 59.80%). Sixty-two subscription-based (62%), 56 open access (56.57%), and only 6 predatory (6.45%) journals suggested, recommended or required study registration. No predatory journals mentioned the Enhancing the Quality and Transparency of health Research (EQUATOR) Network, whereas about a quarter (49/195) of presumed legitimate journals did so.

### Publication model, fees, and copyright

We assessed whether journals made any indication about accessibility, fees, and copyright (Table 9). Forty-two (42.00%) subscription-based journals indicated being partially open access in some capacity (e.g., hybrid or delayed access), with the remainder not mentioning open access. Almost all (*n* = 95, 95.00%) subscription-based journals indicated that there was a subscription charge. Eighty-three potential predatory (89.25%) and 94 open access (94.95%) journals claimed to be open access (presumed to be full, immediate open access as no qualification regarding partial or delayed access was stated). For the five (5.05%) open access journals that did not specifically indicate being open access, all had content that was free to access (we did not investigate this further). Subscription-based journals and open access journals seemed to collect revenue from a range of sources (Table 9), while predatory journals appeared to mainly collect revenues from APCs (*n* = 73, 78.49%) and to a lesser extent, subscription fees (*n* = 13, 13.98); in 14 predatory journals (15.05%), no sources of revenue (including an APC) could be found. Of journals listing an APC, the median fee (USD) was $100 ($63–$150) in predatory journals (*n* = 59), $1866 ($800–$2205) in open access journals (*n* = 70), and $3000 ($2500–$3000) in subscription-based hybrid journals (*n* = 44). Almost 90% of all journals indicated which party retained copyright of published work. Explicit statements that authors retained copyright were present in 68.09% (*n* = 64) of open access journals, 36.78% (*n*2 = 32) of the time in subscription-based journals, and in only 12% (*n* = 9) of predatory journals.

## Discussion

This study demonstrates that our sample of potential predatory journals is distinct in some key areas from presumed legitimate journals and provides evidence of how they differ. While criteria have been proposed previously to characterize potential predatory journals [7], measuring each journal against a long list of criteria is not practical for the average researcher. It can be time consuming and some criteria are not straightforward to apply, as we have learned during this study. For instance, whether or not the listed editors of a journal are real people or have real affiliations with a journal is quite subjective to assess. Another example pertains to preservation and permanent access to electronic journal content. We found that not all presumed legitimate journals made explicit statements about this; however, we know that in order to be indexed in MEDLINE, a journal must “*Have an acceptable arrangement for permanent preservation of, and access to, the content*” [17].

From our findings, we have developed a list of evidence-based, salient features of suspected predatory journals (Table 10) that are straightforward to assess; we describe them further below. We recognize that these criteria are likely not sensitive enough to detect all potentially illegitimate, predatory journals. However, we feel they are a good starting point.

**Table 10:** Salient characteristics of potential predatory journals

1.	The scope of interest includes non-biomedical subjects alongside biomedical topics
2.	The website contains spelling and grammar errors
3.	Images are distorted/fuzzy, intended to look like something they are not, or which are unauthorized
4.	The homepage language targets authors
5.	The Index Copernicus Value is promoted on the website
6.	Description of the manuscript handling process is lacking
7.	Manuscripts are requested to be submitted via email
8.	Rapid publication is promised
9.	There is no retraction policy
10.	Information on whether and how journal content will be digitally preserved is absent
11.	The Article processing/publication charge is very low (e.g., < $150 USD)
12.	Journals claiming to be open access either retain copyright of published research or fail to mention copyright
13.	The contact email address is non-professional and non-journal affiliated (e.g., @gmail.com or @yahoo.com)

### Non-biomedical scope of interest

We found that predatory journals tend to indicate interest in publishing research that was both biomedical and non-biomedical (e.g., agriculture, geography, astrophysics) within their remit, presumably to avoid limiting submissions and increase potential revenues. While legitimate journals may do this periodically (we did not assess the scope of presumed legitimate biomedical journals), the topics usually have some relationship between them and represent a subgroup of a larger medical specialty (e.g., Law and Medicine). Authors should examine the scope and content (e.g., actual research) of the journals they intend to publish in to determine whether it is in line with what they plan to publish.

### Spelling and grammar

The home page of a journal’s website may be a good initial indicator of their legitimacy. We found several homepage indicators that may be helpful in assessing a journal’s legitimacy and quality. The homepages of potential predatory journals’ websites contained at least 10 times more spelling and grammar errors than presumed legitimate journals. Such errors may be an artefact of foreign language translation into English, as the majority of predatory journals were based in countries where a non-English language is dominant. Further, legitimate publishers and journals may be more careful about such errors to maintain professionalism and a good reputation.

### Fuzzy, distorted, or potentially unauthorized image

Potential predatory journals appeared to have images that were low-resolution (e.g., fuzzy around the edges) or distorted ‘knock-off’ versions of legitimate logos or images.

### Language directed at authors

Another homepage check authors can do is to examine the actual written text to gauge the intended audience. We found that presumed legitimate journals appear to target readers with their language and content (e.g., highlighting new content), whereas potential predatory journals seem to target prospective authors by inviting submissions, promising rapid publication, and promoting different metrics (including the Index Copernicus Value).

### Manuscript submission and editorial process/policies

Authors should be able to find information about what happens to their article after it is submitted. Potential predatory journals do not seem to provide much information about their operations compared to presumed legitimate journals. Furthermore, most potential predatory journals request that articles be submitted via email rather than a submission system (e.g., Editorial Manager, Scholar One), as presumed legitimate journals do. Typically, journals have requirements that must be met or checked by authors or the journal during submission (e.g., declaration of conflicts of interest, agreement that the manuscript adheres to authorship standards and other journal policies, plagiarism detection). When a manuscript is submitted via email, these checks are not automatic and may not ever occur. Authors should be cautious of publishing in journals that only take submissions via email and that do not appear to check manuscripts against journal policies as such journals are likely of low quality. In addition, the email address provided by a journal seems to be a good indicator of its legitimacy. Predatory journals seem to provide non-professional or non-academic email addresses such as from providers with non-secured servers like Gmail or Yahoo.

### Very low APC and inappropriate copyright

Finally, authors should be cautious when the listed APC of a biomedical journal is under $150 USD. This is very low in comparison to presumed legitimate, fully open access biomedical journals for which the median APC is at least 18 times more. Hybrid subscription journals charge 30 times the amount of potential predatory journals to publish and make research openly accessible. It has been suggested that hybrid journals charge a higher fee in order to maintain their ‘prestige’ (e.g., journals can be more selective about their content based on who is willing to pay the high fee) [18]. On the contrary, extremely low APCs may simply be a way for potential predatory journals to attract as many submissions as possible in order to generate revenue and presumably to build their content and reputation. Evidently, the APC varies widely across journals, perhaps more than any other characteristic we measured. Journal APCs are constantly evolving and increasing requirements by funders to make research open access may have a drastic impact on APCs as we know them over the coming years.

Researchers should be trained on author responsibilities, including how to make decision about where to publish their research. Ideally, authors should start with a validated or ‘white’ list of acceptable journals. In addition to considering the items listed in Table 10 in their decision-making, tools to guide authors through the journal selection process have started to emerge, such as ThinkCheckSubmit (http://thinkchecksubmit.org/). Recently, COPE, OASPA, DOAJ, and WAME produced principles of transparency against which, among other measures, DOAJ assesses journals in part, before they can be listed in the database (https://doaj.org/bestpractice). We also encourage researchers to examine all journals for quality and legitimacy using the characteristics in Table 10 when making a decision on where to submit their research. As the journal landscape changes, it is no longer sufficient for authors to make assumptions about the quality of journals based on arbitrary measures, such as perceived reputation, impact factor, or other metrics, particularly in an era where bogus metrics abound or legitimate ones are being imitated.

This study examined most of Beall’s criteria for identification of predatory publishers and journals together with items from the COPE and OASPA. While many of the characteristics we examined were useful to distinguish predatory journals from presumed legitimate journals, there were many that do not apply or that are not unique to predatory journals. For instance, defining criteria of predatory journals [4] suggest that no single individual is named as an editor and that such journals do not list an editorial board. We found that this was not the case in over two thirds of predatory journals and, in fact, a named EIC could not be identified for 26 (13.07%) of the presumed legitimate journals in our sample. Such non evidence-based criteria for defining journals may introduce confusion rather than clarity and distinction.

The existing designation of journals and publishers as predatory may be confusing for other reasons. For instance, more than one presumed-legitimate publisher has appeared on Beall’s list [19]. In October 2015, Frontiers Media, a well-known Lausanne-based open access publisher, appeared on Beall’s List [20]. Small, new, or under-resourced journals may appear to have the look and feel of a potential predatory journal because they do not have affiliations with large publishers or technologies (e.g., manuscript submission systems) or mature systems and the features of a legitimate journal. This is in line with our findings that journals from low-resourced (LMIC) countries were more often in the potentially predatory group of journals than either of the presumed-legitimate journal arms. However, this does not imply that they are necessarily predatory journals.

Another limitation is that the majority of the open access biomedical journals in our sample (95%) charged an APC, while generally many open access journals do not. May 2015 was the last time that the DOAJ provided complete information regarding APCs of journals that it indexes (fully open access, excluding delayed or partial open access). At that time, approximately 32% of journals charged an APC. At the time of writing this article, approximately 40% of medical journals in DOAJ appear to charge an APC. However, these figures do not account for the hybrid-subscription journals that have made accommodations in response to open access, many of which are included in our sample of subscription-based journals. For such journals, our data and that of others [21] show that their fees appear to be substantially higher than either potential predatory or fully open access journals.

#### In context of other research

To the best of our knowledge this is the first comparative study of predatory journal publishing and legitimate publishing models aimed at determining how they are different and similar. Previously, Shen and Björk [22] examined a sample of about 5% of journals listed on Beall’s List for a number of characteristics, including three that overlap with items for which we collected data: APC, country of publisher, and rapidity of (submission to) publishing [22]. In a large part, for the characteristics examined, our findings within the predatory journal group are very similar. For example, Shen and Björk [22] found the average APC for single publisher journals to be $98 USD, which is very similar to our results ($100 USD). They also found that 42% of single predatory journal publishers were located in India, whereas our estimates were closer to 62%. Differences between their study and ours may exist because we focused on biomedical journals while they included all subject areas.

### Limitations

It was not possible to fully blind assessors to study groups since, given the expertise of team members, a minimum knowledge of non-predatory publishers was expected. In addition, we could only include items that could be assessed superficially rather than those requiring in-depth investigations for each journal. Many items can and should be investigated further.

Since some characteristics are likely purposely similar between journals (e.g., journals from all groups claim to be open access and indicate carrying out peer review) [14], and it was difficult to anticipate which, we did not carry out a logistic regression to determine whether characteristics were likely to be associated with predatory or presumed legitimate journals.

## Conclusions

This research initiates the evidence-base illuminating the difference between major publishing models and, moreover, unique characteristics of potential predatory (or illegitimate) journals (Table 10).

The possibility that some journals are predatory is problematic for many stakeholders involved in research publication. Most researchers are not formally trained on publication skills and ethics, and as such may not be able to discern whether a journal is running legitimate operations or not. For early career researchers or for those who are unaware of the existence or characteristics of predatory journals, they can be difficult to distinguish from legitimate journals. However, this study indicates that predatory journals are offering at least 18-fold lower APCs than non-predatory journals, which may be attractive to uninformed authors and those with limited fiscal resources. Assuming that each journal publishes 100 articles annually, the revenues across all predatory journals would amount to at least a $USD 100 million dollar enterprise. This is a substantial amount of money being forfeited by authors, and potentially by funders and institutions, for publications that have not received legitimate professional editorial and publishing services, including indexing in databases.

Established researchers should beware of predatory journals as well. There are numerous anecdotes about researchers (even deceased researchers [23]) who have been put on a journal’s editorial board or named as an editor, who did not wish to be and who were unable to get their names delisted [24]. Aside from this potentially compromising the reputation of an individual that finds him or herself on the board, their affiliation with a potential predatory journal may confer legitimacy to the journal that is not deserved and that has the potential to confuse a naïve reader or author. As our findings indicate, this phenomenon appears to be a clear feature of predatory journals.

In addition to the costs and potential fiscal waste on publication in predatory journals, these journals do not appear to be indexed in appropriate databases to enable future researchers and other readers to consistently identify and access the research published within them. The majority of predatory journals indicated being ‘indexed’ in Google Scholar, which is not an indexing database. Google does not search pre-selected journals (as is the case with databases such as Medline, Web of Science, and Scopus), rather it searches the Internet for scholarly content. Some potentially predatory journals indicate being indexed in well-known biomedical databases; however, we have not verified the truthfulness of these claims by checking the databases. Nonetheless, if legitimate clinical research is being published in predatory journals and cannot be discovered, this is wasteful [25], in particular when it may impact systematic reviews. Equally, if non-peer reviewed, low quality research in predatory journals is discovered and included in a systematic review, it may pollute the scientific record. In biomedicine, this may have detrimental outcomes on patient care.

### Future research

What is contained (i.e., ‘published’) within potential predatory journals is still unclear. To date, there has not been a large-scale evaluation of the content of predatory journals to determine whether research is being published, what types of studies predominate, and whether or not data (if any) are legitimate. In addition, we have little understanding of who is publishing in predatory journals (i.e., experience of author, geographic location, etc.) and why. Presumably, the low APC is an attractive feature; however, whether or not authors are intentionally or unintentionally publishing within these journals is critical to understanding the publishing landscape and anticipate future potential directions and considerations.

The findings presented here can facilitate education on how to differentiate between presumed legitimate journals and potential predatory journals.

## Abbreviations

AIM: Abridged Index Medicus
APC: article processing charge
CONSORT: CONsolidated Standards Of Reporting Trials
COPE: Committee On Publication Ethics
DOAJ: Directory Of Open Access Journals
EIC: editor-in-chief
EQUATOR: Enhancing the QUAlity and Transparency of health Research
ISSN: international standard serial number
JIF: journal impact factor
LMIC: low- or middle-income country
OASPA: Open Access Scholarly Publishers Association
PLOS: Public Library Of Science
PRISMA: Preferred Reporting Items for Systematic reviews and Meta-Analyses
STARD: STAndards for Reporting Diagnostic accuracy
STROBE: STrengthening the Reporting of OBservational studies in Epidemiology
USD: United States Dollar
